# Assessing out-of-pocket expenditures for primary health care: how responsive is the Democratic Republic of Congo health system to providing financial risk protection?

**DOI:** 10.1186/s12913-018-3211-x

**Published:** 2018-06-15

**Authors:** Samia Laokri, Rieza Soelaeman, David R. Hotchkiss

**Affiliations:** 1 Tulane University, School of Public Health and Tropical Medicine, Department of Global Community Health and Behavioral Sciences, 1440 Canal St, Rm 226, New Orleans, LA 70112 USA; 2 Université Libre de Bruxelles, School of Public Health, Research center Health policy and systems - Interntional Health, Brussels, Belgium; 3 Université Libre de Bruxelles, Institute for Interdisciplinary Innovation in Healthcare (I3h), Brussels, Belgium; 4 Tulane university, School of Public Health and Tropical Medicine, Department of Global Health Management and Policy, 1440 Canal St, Ste 1900, New Orleans, LA 70112 USA

**Keywords:** Access to primary health care, Direct cost, Democratic Republic of Congo, Health equity, Health financing reform, health insurance coverage, low-income countries, Outpatient care/cost of ambulatory care, Out-of-pocket expenditure for health, sustainable health system

## Abstract

**Background:**

The goal of universal health coverage is challenging for chronically under-resourced health systems. Although household out-of-pocket payments are the most important source of health financing in low-income countries, relatively little is known about the drivers of primary health care expenditure and the predictability of the burden associated with high fee-for-service payments. This study describes out-of-pocket health expenditure and investigates demand- and supply-side drivers of excessive costs in the Democratic Republic of Congo (DRC), a central African country in the midst of a process of reforming its health financing system towards universal health coverage.

**Methods:**

A population-based household survey was conducted in four provinces of the DRC in 2014. Data included type, level and utilization of health care services, accessibility to care, patient satisfaction and disaggregated health care expenditure. Multivariate logistic regressions of excessive expenditure for outpatient care using alternative thresholds were performed to explore the incidence and predictors of atypically high expenditure incurred by individuals.

**Results:**

Over 17% **(**17.5%) of individuals living in sample households reported an illness or injury without being hospitalized. Of 3341 individuals reporting an event in the four-week period prior to the survey, 65.6% sought outpatient care with an average of one visit (SD = 0.0). The overall mean expenditure per visit was US$ 6.7 (SD = 10.4) with 29.4% incurring excessive expenditure. The main predictors of a financial risk burden included utilizing public services offering the complementary benefit package, dissatisfaction with care received, being a member of a large household, expenditure composition, severity of illness, residence and wealth (*p* < .05). The insured status influenced the expenditure level, with no association with catastrophe. Those who did not seek care when needed reported financial constraints as the major reason for postponing or foregoing care. Wealth-related inequities were found in service and population coverage and in out-of-pocket payment for outpatient care.

**Conclusion:**

Burdensome expenditure for primary care and its key drivers are of utmost importance. Forthcoming health financing reform agendas must incorporate a strategy for getting data used in the design of financial risk protection. Realizing equitable and efficient access to outpatient care is a vital ingredient for sustainable health systems.

## Background

Making progress towards universal health coverage (UHC) goals within chronically under-resourced health systems is challenging. In many low-income countries, household out-of-pocket payments are the most important source of health financing. Health facilities mostly rely on user fees to finance their operating costs, health staff salaries, and target viable and quality structures [1]. Reliance on out-of-pocket health financing does not constitute a planned health financing strategy per se, but rather a coping mechanism which attempts to ensure a sustainable model of health care delivery. Nevertheless, such a mechanism may put households at tangible risk of catastrophic expenditures for accessing primary and other types of care [2, 3].

In the Democratic Republic of the Congo (DRC), per capita health expenditure remains low and largely below what other low-income countries have invested. While the country is in the process of reforming health financing in order to make progress towards UHC goals, the fragile context of the DRC recalls the current inability of the state to widely deliver quality health care as needed, and to regulate the health sector in order to protect primary care users from excessive fee-for-service payments [4–6]. With 0.7% of the GDP and 4% of the state budget, the financing gap for health is considerable as the country aims to achieve the UHC strategic objectives. Recent evidence suggests that, in a context of chronic reliance on direct payments, households are constantly threatened by the risk of unpredictable and catastrophic payments for health with a genuine danger of suppressing households’ nonmedical consumption [7] particularly among the poor [8, 9]. For Congolese households, the threat of catastrophic expenditure on primary health care is therefore critical, as they finance 40% of health services, 90% of which is made through direct payments [10]. This resource-constrained situation is concerning and yet there is little evidence on the drivers of primary health care expenditure and the predictability of the burden associated with high fee-for-service payments.

In designing an effective UHC strategy, the country must take into account evidence available on persistent inequalities and prioritize measures to alleviate the economic burden of illness or injury that are managed by the front-line care providers. At the same time, the quality of services is also a concern. Recent evidence on the availability and operational capacity of health facilities in the DRC highlight a series of constraints that may undermine the path to UHC [11, 12]. Especially in rural areas, the availability of well-trained human resources for health does not meet international standards, distance to care continues to be a significant barrier, and large stock-outs of essential drugs are common in most health facilities (e.g. rehydration salts only in 2% of health facilities, antibiotics only 53% of health facilities, etc.). In terms of equity, evidence has confirmed the presence of disparities by type of institution, managerial body and urban-rural residence.

The availability of population-based household surveys are needed to estimate the extent of financial risk protection and the poverty impact of illness for specific interventions, such as primary health care services, is very limited [13].

In step with the global agenda for universal access to health, several efforts have been made in the DRC. Constitutionally, the country has for the last two years been engaged in a process of transferring competencies to the provinces in several areas, comprising that of primary health care organization. In December 2014, the adoption of resolutions and recommendations from the General Assembly of the National Steering Committee of the Health Sector [Comité National de Pilotage du Secteur de la Santé – CNP-SS] effectively decentralized the health system and established major health reforms, including the implementation of Provincial Health Divisions [Divisions provinciales de la santé – DPS] [14]. New public health legislation is being examined by the Parliament and discussed in the National Assembly and the country intends to adopt a potentially groundbreaking UHC bill that would significantly impact the health sector. In particular, several health coverage schemes are under consideration (i.e., two separate public and private not-for-profit coverage schemes and a national fund for UHC). Key stakeholders (i.e., the EU-Luxembourg-WHO Universal Health Coverage Partnership) support the policy dialogue on national health policies and are committed to strengthening the UHC partnership’s program. Along with other countries, the DRC agreed on a revised roadmap toward UHC in Brazzaville in March 2016. Recently, a key UHC policy note drew attention to a deficient coverage in the country and to the fact that contribution of informal sector households cannot meet the financing requirements. [15]. To date, for the vast majority of the population, the only mechanism for sharing health risks is voluntary community-based health insurance. The potential for the growth of existing fiscal space is being explored to mobilize sufficient funds to improve access to health for all. However, outpatient-focused research is needed to build well-designed delivery systems that both improve supply and demand of basic health care services [16]. In this regard, the Alma-Ata Declaration 40th anniversary revived interest in primary care research [17]. On this occasion, the Lancet challenges the scientific community to provide new evidence and support the revised Declaration 2.0 [17, 18]. In sum, despite strong political commitments, the lack of empirical evidence, which was reported as a matter of concern to UHC stakeholders, impairs the path to a better tomorrow.

The aim of this study was to gain a deeper understanding of the poverty impact of using essential care services in selected provinces in the DRC. We sought to inform the evidence base regarding the policy and technical challenges inherent to developing financial risk protection strategies. We assessed the poverty impact of primary health care expenditure using a two-pronged approach. First, we estimated itemized individual-level medical and nonmedical expenses associated with outpatient health care utilization in the DRC. This approach used both descriptive statistics, as well as concentration indices to describe wealth-related inequities in utilization and expenditure, along with regional variations. Second, we used multivariate modeling techniques to assess the major components and predictors of direct expenditure that may constitute a catastrophic burden on households. The results of the study are hoped to be useful in refining the national health financing policy in the DRC.

## Methods

### Study design

This study presents the findings from a 2014 baseline survey conducted as part of an impact evaluation of IMA World Health’s Accès aux Soins de Santé Primaires (ASSP) project in the DRC. The survey consisted of a household survey and a linked health facility survey. To ensure comparability with information from the Demographic and Health Survey (DHS), questionnaires and survey procedures consistent with the standardized methodology and guidelines set by the MEASURE DHS program were developed [19]. ASSP is a United Kingdom Department for International Development-funded health systems strengthening project working in 52 health zones in Equateur, Orientale, Kasai-Occidental, Kasai-Oriental and Maniema provinces. The surveys collected information on a variety of topics including health care utilization, out-of-pocket payments, prices of select medical procedures at government and private health facilities, health outcomes, client satisfaction, household assets, as well as individual- and household-level factors associated with health outcomes and health care utilization.

### Outpatient survey

Household representatives were asked if each member of their household had been sick or injured in the four weeks prior to the survey. Individuals who reported an event without being hospitalized were eligible for the outpatient survey. They were asked to report on outpatient care visits from public and private health care providers, pharmacists or traditional healers. These include outpatient family planning visits, prenatal and postnatal care, and monitoring of child health. Respondents were asked both the total and itemized out-of-pocket expenditure for each episode of illness or injury (including informal items and nonmedical items such as transportation). For those who did not seek care, reasons to postpone or forego care were investigated. Survey instruments included structured questionnaires administrated to household representatives who were encouraged to consult with other members to ensure complete responses. Questionnaires were translated into the local languages of Lingala, Swahili, and Tshiluba.

### Sampling and data collection procedures

A two-stage sampling strategy was used. In the first stage, three sampling areas were defined as the provinces Orientale and Maniema combined, Kasai-Occidental and Kasai-Orientale combined (the latter was included in the study in order to provide the ability to select matched comparison areas for intervention areas in Kasai-Occidental), and Equateur. A total of 35 villages per sampling area were selected in both intervention areas and matched comparison areas using probability proportional to size (PPS). In the second stage, after fully enumerating villages, systematic random sampling was used to select a constant number of 20 households per village to obtain the necessary sample size of 700 households in each sampling area for an expected total of 4200 households. Once households were selected, the head of household or another household member was interviewed. The survey yielded a 94% response rate.

### Analytical framework and variables definitions

**Fig. 1:**
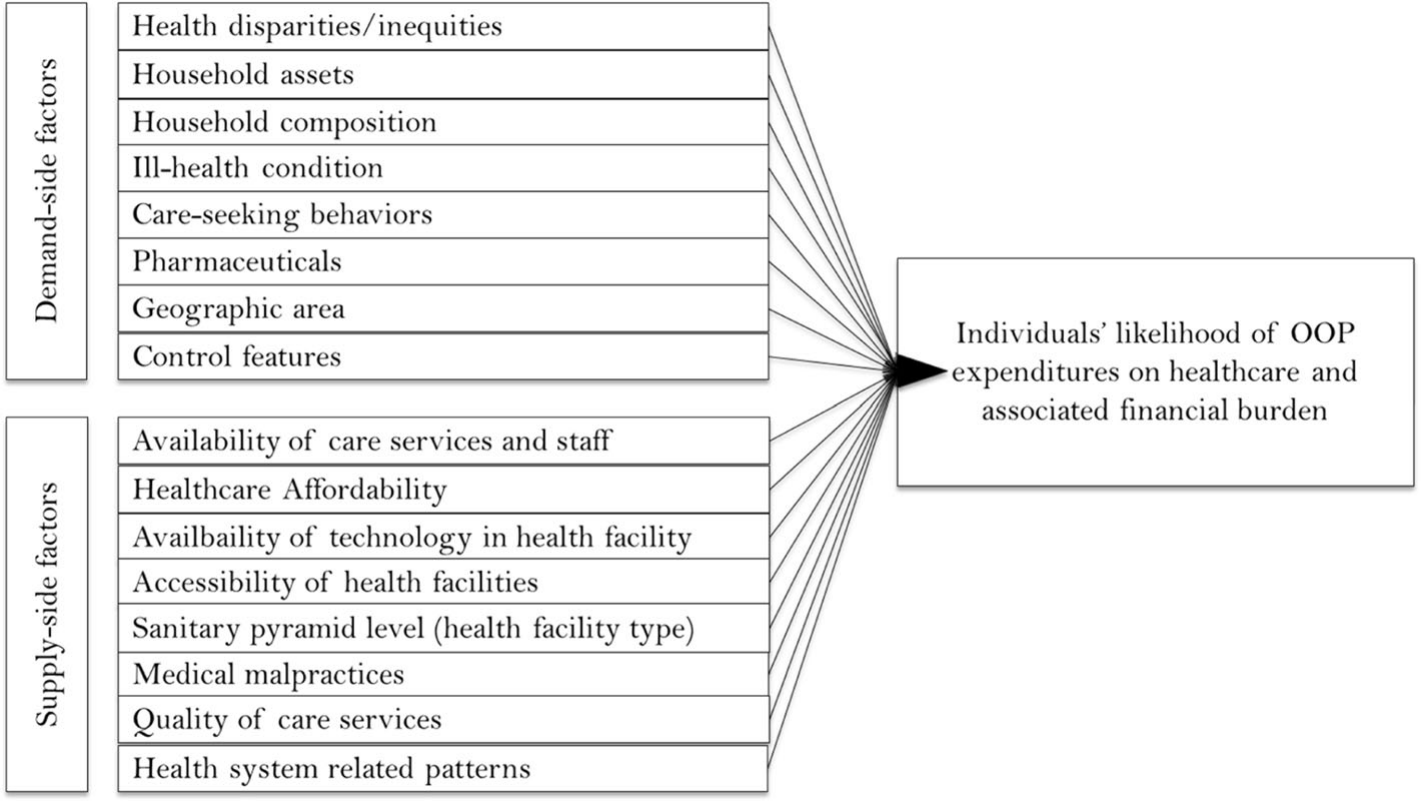
A multidimensional framework of predictors influencing the burden of patients’ out-of-pocket expenditure (OOPs)

The primary study variable was the overall reported out-of-pocket expenditures (OOPs) for outpatient recorded in Congolese Francs (US$ 1 = 900 Congolese Francs). Total reported amounts were used and the sum of itemized expenditures were imputed to the total reported for missing values. Based on a review of previous studies [20–23], a framework of potential drivers of OOPs was used to consider demand- and supply-side explanatory variables (Fig. 1). Characteristics related to the demand for care consisted of individual demographic characteristics, household composition and status, socio-economic factors, illness, or care-seeking behavior while those related to the supply of care services referred to the availability and perceived quality of care, and affordability of care. We considered the influence of the above-listed factors on the likelihood of high expenditures and financial burden due to outpatient care utilization. Independent variables included various indices computed using Principal Component Analysis (i.e. wealth, health facility scores, patient satisfaction). For instance, a composite index of patient satisfaction with care services received was used to approximate the perceived quality of outpatient services. In addition, the reported days lost for the period the person was incapacitated were used to reflect severity of illness.

### Statistical analysis

Our aim was to estimate the levels and drivers of primary OOPs in DRC, while also examining wealth-related inequalities in health care utilization and expenditures. Descriptive analysis was used for characterizing the study population. This study investigated the relationship between the extensive list of risk factors and the dependent variable. To ensure robustness of findings, we investigated univariate logistic models and estimated multilevel multivariate logistic regression. The multilevel model took into account nesting of individuals within households and included information from the health facility where they tended to most likely seek care.

The response variable categorizing OOPs was operationalized as a dichotomous variable using three alternative thresholds: “high expenditure” for the main model, “medium-high expenditure” and “extremely high expenditure” in the models used for sensitivity analyses. “High expenditure” was defined as having expenditure greater than or equal to two times the median, while “medium-high expenditure” was defined as having expenditure greater than or equal to the median. The final threshold, “extremely high expenditure,” was defined as having expenditure greater than or equal to three times the median. We validated our model using a variety of sensitivity and scenario analyses. Multiple sets of predictors were used to determine the best fitting model. Therefore, various methods including principal component analysis (PCA) for computing the covariates were used to test sensitivity. Further, multicollinearity analysis was conducted to test the correlation of the error terms of the independent variables. In addition, for each set of covariates, models were estimated using each of the three identified thresholds of excessive overall reported OOPs.

Student’s t test was performed to assess differences between two means. When needed, the Mann-Whitney U test was used. Univariate logistic regression measured associations between the potential predictors and the response variable. Either χ^2^ test or Fisher’s exact test was used to test the degree of association of categorical variables. We included covariates with *p* < 0.20 in the multivariate models. The logit specification was used to estimate the relative effects of explanatory variables for the multivariate regression analyses. Crude and adjusted odds of incurring medium-high, high, and extremely high OOPs, and related *p*-values, were analyzed. Pearson’s correlation was used to explore the correlation between determinant and response variable. The analysis was conducted using Stata 14.0 (StataCorp. 2015. Stata Statistical Software: Release 14. College Station, TX: StataCorp LP).

## Results

### Characteristics of the study population

Among households selected for the survey, 98.6% (*n* = 4120) were successfully interviewed. Among individuals living in these households, 17.5% were reported to have been sick or injured in the four weeks prior to interview. Table 1 describes the study population.

**Table 1:** Characteristics of the study population – Percent distribution or mean (SD) of individuals in sampled areas by selected background characteristics

Characteristics	Study areas			Total
	Equateur	Kasai-Occidental / Oriental	Maniema / Orientale	
Setting				
Rural	93%	91%	92%	92%
Urban	7%	9%	8%	8%
Gender				
Female	54%	55%	57%	56%
Male	46%	45%	43%	44%
Age (in years)				
< 5	33%	28%	31%	29%
5–14	17%	23%	24%	22%
15–24	10%	11%	9%	11%
25–34	13%	11%	7%	10%
35–44	7%	8%	7%	8%
45–54	8%	7%	10%	8%
55+	11%	12%	11%	11%
Average (SD)	21.94 (1.11)	22.78 (0.94)	21.07 (1.51)	22.18 (0.72)
Wealth quintile				
Low	12%	40%	3%	26%
Low Middle	35%	20%	11%	19%
Middle	22%	14%	28%	19%
High Middle	18%	13%	31%	19%
High	13%	13%	27%	17%
Household size				
1–3	18%	17%	14%	16%
4–8	66%	66%	74%	69%
> 8	16%	16%	12%	15%
Average (SD)	5.97 (0.1)	6.15 (0.1)	5.9 (0.1)	6.1 (0.1)
Head of household	14%	15%	7%	13%
Highest education of household head				
None completed	13%	20%	6%	15%
Primary	32%	25%	25%	26%
Secondary	54%	51%	63%	55%
Tertiary	1%	4%	5%	4%
Time to access outpatient care				
≥ 30 min	96%	85%	96%	89%
< 30 min	4%	15%	4%	11%
Distance of facility from the village (in km)	9.3 (0.9)	7.9 (0.5)	5.5 (0.3)	7.4 (11.8)

*All estimates are weighted*

### Service and population coverage and quality

Among individuals with illness or injury, 65.6% sought care (all-cause) at least once (Table 2). On average, individuals had one outpatient visit per episode of illness or injury. A large proportion of users sought care at a public sector facility, primarily at general reference hospitals and health centers. Comparatively, individuals sought care in a limited and very limited way respectively from private and informal providers. A total of 11.8% of clients reported being satisfied with the care received while 57.7% claimed at least three reasons for dissatisfaction. In particular, half of the care users reported being satisfied with respect to the provider’s skill and waiting time. A lesser proportion reported being satisfied with the provider’s explanation, the equipment in the health facility and the drug supply. Besides, very few (3.8%) individuals benefitted from the coverage of a health insurance scheme. Of those covered by a scheme, the insurer type (mutual health organization or insurance through employer) greatly varied across the study areas.

**Table 2:** Service and population coverage – Percent distribution or mean (SD) of individuals in sampled areas

Service and population coverage	Study areas			Total
	Equateur	Kasai-Occidental /Oriental	Maniema /Orientale	
Sought outpatient care				
All types of care	78.0%	62.9%	65.8%	65.6%
Number of outpatient visits				
Charged visits, per household	1.5 (0.0)	1.7 (0.0)	1.6 (0.0)	1.6 (0.0)
Charged visits, per capita	0.7 (0.0)	0.6 (0.0)	0.6 (0.0)	0.6 (0.0)
Free-of-charge visits, per capita	0.3 (0.0)	0.4 (0.0)	0.4 (0.0)	0.4 (0.0)
Any, per capita	1.0 (0.0)	1.0 (0.0)	1.0 (0.0)	1.0 (0.0)
Outpatient visits, any				
1 visit, per household	61.0%	55.3%	53.3%	55.5%
2 to 9 visits, per household	39.0%	44.7%	46.7%	44.5%
1 visit, per capita	98.1%	99.3%	97.2%	98.5%
2 to 3 visits, per capita	1.9%	0.7%	2.8%	1.5%
Primary use of care				
Public medical sector	76.6%	59.5%	59.1%	61.9%
Private medical sector	8.1%	27.3%	34.4%	26.6%
Other source	15.2%	13.2%	6.6%	11.5%
Type of facility: Public medical sector				
General Reference hospital	36.8%	63.9%	47.5%	55.8%
Health centre	49.3%	30.9%	9.2%	26.8%
Reference health center	11.0%	0.2%	32.3%	10.9%
Secondary hospital	0.6%	2.0%	0.0%	1.2%
Health post	0.0%	0.0%	1.7%	0.5%
Type of facility: Private sector				
Hospital/private clinic	0.6%	3.0%	7.8%	4.1%
Specialised clinic	0.0%	0.0%	1.5%	0.4%
Other private sector	1.7%	0.0%	0.0%	0.2%
Satisfaction with care received				
with provider skill	65.1%	47.1%	55.8%	51.8%
with waiting time	56.3%	46.8%	54.4%	50.2%
with provider explanation	45.6%	33.8%	41.3%	37.4%
with health facility equipment	47.3%	28.6%	36.5%	33.2%
with drug supply	46.2%	20.6%	16.6%	22.5%
with all five items	28.4%	10.9%	6.7%	11.8%
Health insurance coverage				
Any type of insurance, of which:	3.8%	0.2%	1.4%	1.0%
*Mutual health organization*	87.9%	14.3%	41.0%	58.4%
*Employer-offered plan*	12.1%	72.3%	58.7%	39.5%
*Other commercial plan*	0.0%	13.4%	0.3%	2.1%
None	96.2%	99.8%	98.6%	99.0%
Top reasons for delaying care				
Financial constraints	76.7%	76.4%	44.0%	65.9%
Self-medication	11.7%	17.8%	3.1%	12.3%
Distance to facility	1.8%	0.0%	34.2%	11.4%

*All estimates are weighted; [*] *] more than 10% missing*

Individuals who did not seek care when needed (34.4%) reported at least one reason for postponing or foregoing care. The reasons most frequently reported were financial constraints, preference for self-medication, and distance to the facility.

### Out-of-pocket expenditures (OOPs) and financial risk protection for primary care

Among individuals who sought outpatient care (*n* = 2412), 89.9% had non-zero monetary expenditures and 0.9% reported in-kind payments (Table 3). Among those who spent money for care (*n* = 2168), the mean overall OOPs for outpatient care was US$ 6.8 (0.4). The most important expense item was fees for drugs and medicines (62.3%), followed by fees for consultation (32.7%). Nonmedical items such as travel and food accounted for 2.3% of total payments. The average amount spent in the public or private sector was US$ 7.0, while in the informal sector, it was US$ 3.9. Individuals who spent less than the median OOPs (i.e., less than US$ 3.0) (37.7%) spent an average of US$ 1.3 (0.7), whereas for the 21.7% in the extremely high group, the average expenditure was US$ 21.7 (15.3). Overall, expenditures were higher among the 3.9% of individuals with insurance coverage compared to uninsured individuals (US$ 8.5 versus US$ 6.7). Regardless of wealth quintile, the expenditure averaged US$ 19.39, which is even much higher, under employer-offered plan compared to US$ 5.22 under community-based insurance versus.

**Table 3:** Levels and shares of total out-of-pocket expenditure (mean OOPs) for outpatient care

	Mean	SD	Frequency	Mean share of total OOPs	SD
Individuals who used care (n_1_ = 2412)					
Aggregate costs (mean, sd, n, %)					
Overall reported cost	6.20	0.35	100.0%	na^a^	na
Care users who spent >USD 0 (n_2_ = 2168)					
Aggregate costs (mean, sd, n, %)					
Overall reported OOPs	6.77	0.37	100.0%	na	na
Overall reported cost, per visit	6.61	0.37	100.0%	na	na
Itemized OOPs					
Total medical cost, per visit	6.37	0.38	100.0%	97.7%	0.9%
*Drugs and medicines*	4.90	0.34	99.7%	62.3%	8.9%
*Consultation*	2.33	0.27	74.9%	32.7%	9.0%
*Laboratory tests*	1.05	0.12	38.5%	1.4%	0.5%
*X-ray*	0.63	0.26	14.1%	0.8%	0.4%
*Medical products*	0.02	0.01	13.2%	0.5%	0.2%
Total non-medical cost, per visit	2.71	0.58	7.6%	2.3%	0.9%
*Transportation*	0.45	0.22	15.0%	0.7%	0.3%
*Other*	0.64	0.12	19.9%	1.6%	0.7%
Health sector					
Public medical	7.00	9.39	67.3%	na	na
Private medical	7.30	12.76	25.2%	na	na
Informal	3.93	8.28	7.5%	na	na
Excessive OOPs analysis groups					
Low (<p50)	1.31	0.69	37.7%	na	na
Medium-high (≥p50)	3.89	0.82	27.7%	na	na
High (≥ 2 *times* p50)	6.87	0.80	13.5%	na	na
Extremely high (≥ 3 *times* p50)	21.66	15.28	21.1%	na	na
Setting					
Rural	6.40	9.61	88.1%	na	na
Urban	10.88	16.15	11.9%	na	na
Study areas					
Equateur	4.44	6.02	26.2%	na	na
Kasai-Occidental / Oriental	5.82	10.25	36.8%	na	na
Maniema / Orientale	9.69	11.58	37.0%	na	na
Gender					
Female	7.07	10.58	55.1%	na	na
Male	6.26	10.07	44.9%	na	na
Health insurance status					
Covered, any scheme	8.53	16.28	3.9%	na	na
*Mutual health organization*	5.22	7.80	86.6%	na	na
*Employer-offered plan*	19.39	29.23	12.2%	na	na
*Other commercial plan*	6.00	na	1.2%	na	na
Uncovered	6.70	10.28	96.1%	na	na

All estimates are weighted and reported for individuals who spent money for outpatient care

^a^*na* Not applicable

### Equity in care utilization, coverage and out-of-pocket payments

**Fig. 2:**
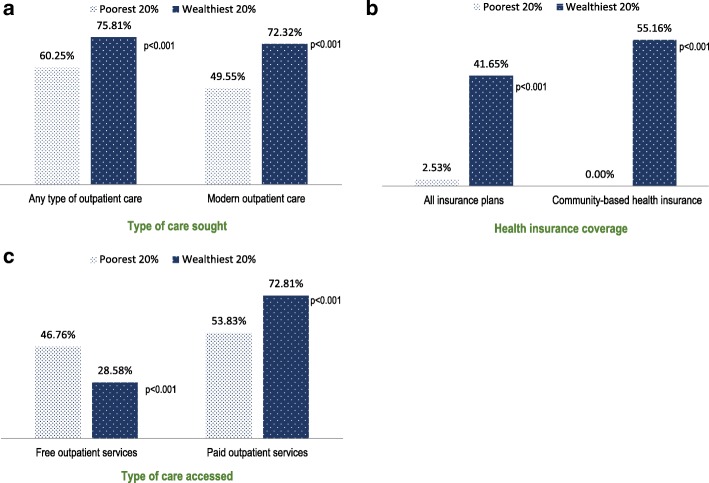
Equity gaps in outpatient care utilization according to ability to receive care for patients in need of outpatient care, health insurance coverage and type of care accessed. **a**) Type of care sought. **b**) Health insurance coverage. **c**) Type of care accessed

We found evidence of wealth-related disparities in outpatient care utilization, coverage and health payments. Only 60.3% of individuals in the poorest wealth quintile who reported illness in the past four weeks sought any type of care compared to 75.8% among those in the wealthiest quintile (*p* < 0.001) (Fig. 2a). The rich-poor gap in care utilization was even greater among those who sought modern medical care, with 49.6% of individuals in the poorest households and 72.3% of those in the wealthiest households seeking care at a health facility (*p* < 0.001). More specifically, the percent of care users covered by an insurance scheme was 41.7% among the wealthiest of the population compared to only 2.5% of the poorest group (Fig. 2b). Compared to the wealthiest group of care users, the poorest group proportionally also resorted more to free-of-charge visits and less to visits for which fees are charged to the patients (*p* < 0.001) (Fig. 2c).

**Fig. 3:**
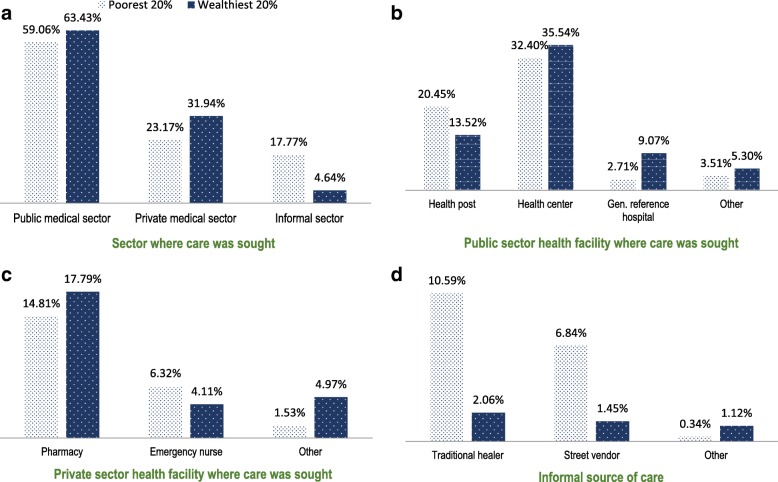
Equity gaps in outpatient care utilization according to health sector and providers’ type in each health sector. **a**) Sector where care was sought. **b**) Public sector health facility where care was sought. **c**) Private sector health facility where care was sought. **d**) Informal source of care

In addition, utilization of care was greater among care users belonging to the wealthiest quintile (Fig. 3a). Compared to the poorest quintile, they were more likely to use more specialized care services, such as the complementary benefit package available at general reference hospitals or to buy drugs in private pharmacies (Figs. 3b and c). In contrast, the poorest care users were more likely to use informal services such as traditional healers and street vendors (Fig. 3d).

**Fig. 4:**
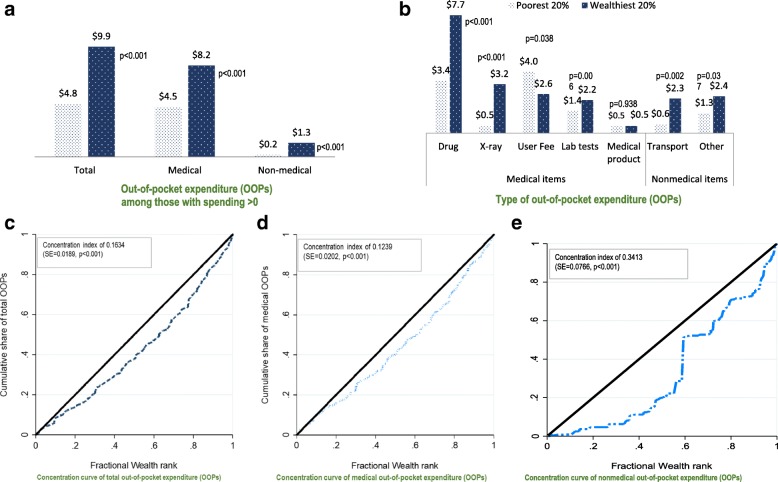
Equity gaps in out-of-pocket expenditure (mean OOPs) for outpatient care. **a**) Out-of-pocket expenditure (OOPs) among those with spending >0. **b**) Type of out-of-pocket expenditure (OOPs). **c**) Concentration curve of total out-of-pocket exepnditure (OOPs). **d**) Concentration curve of medical OOPs. **e**) Concentration curve of nonmedical OOPs

Overall reported average OOPs ranged from US$ 4.8 (SD = 6.6) for care users belonging to the poorest quintile of the population to US$ 9.9 (SD = 12.7) for those belonging to the wealthiest quintile (*p* < 0.001) (Fig. 4a). In all cases, wealth contributed to inequity in health expenditure for primary care with a ratio of 1 to 6 for the maximum amounts spent by the wealthiest compared to the poorest group (Fig. 4b). The direct burden of OOPs showed a pro-rich distribution with a concentration index of 0.16 (SE = 0.02, *p* < 0.001) (Fig. 4c). Concentration curves for nonmedical expenditure lied further below the 45° line set for equity (Fig. 4e), suggesting a higher inequity favoring the rich than for medical expenditure (Fig. 4d).

### Univariate logistic regression analysis

Among individuals incurring high levels of OOPs, the highest proportion of individuals (32.6%) were in the wealthiest quintile compared to other wealth quintiles (p < 0.001) (Table 4). In Equateur, the percent of incurring high expenditure was lowest at 9.3% compared to around 45% of individuals in the remaining two sampling areas. The percent was much higher when children under 5 years old used care services (70.5% versus 29.5%). The percent was also highest among public sector facility users at 71.6%, compared to 23.8 and 4.7% in the private and informal sectors, respectively.

**Table 4:** Univariate logistic regression analysis including the association of independent variables with the likelihood of incurring excessive out-of-pocket expenditure (OOPs) for outpatient care

Determinant	% (*n*) of the determinants		Rate of high OOPs in subgroups (%)	Crude OR [95% CI]	*P*-value	Pearson Chi2
Wealth quintile						< 0.001^***^
Poorest	23.80 (67,911)		18.10	1		
Poorer	19.50 (55,595)		17.80	1.28 [0.76–2.17]	0.356^ns^	
Middle	18.70 (53,245)		16.40	1.21 [0.65–2.28]	0.546^ns^	
Wealthier	17.60 (50,255)		15.00	1.16 [0.58–2.32]	0.666^ns^	
Wealthiest	20.30 (57,793)		32.60	3.12 [1.82–5.37]	< 0.001^***^	
Highest education of household head						0.776^ns^
None	13.40 (38,038)		13.90	1.06 [0.58–1.95]	0.849^ns^	
Primary	24.80 (70,631)		23.20	0.91 [0.6–1.4]	0.682^ns^	
Secondary	57.30 (163,166)		57.20	1		
Tertiary	4.50 (12,754)		5.60	1.41 [0.71–2.83]	0.326^ns^	
Owns a means of transportation						0.882^ns^
No	62.10 (176,942)		62.60	1		
Yes	37.90 (107,878)		37.40	0.97 [0.67–1.41]	0.882^ns^	
Large household size (≥6)						0.056^ns^
No	63.10 (179,630)		56.20	1		
Yes	36.90 (105,191)		43.80	1.51 [0.99–2.31]	0.057^ns^	
Province						< 0.001^***^
Equateur	14.40 (40,978)		9.30	0.28 [0.14–0.56]	< 0.001^***^	
Kasaï Occidental and Oriental	56.30 (160,426)		45.70	0.38 [0.18–0.8]	0.011^*^	
Maniema /Orientale	29.30 (83,416)		45.00	1		
Gender						0.281^ns^
Female	54.70 (153,660)		56.70	1		
Male	45.30 (127,359)		43.30	0.89 [0.72–1.1]	0.281^ns^	
Head of household						0.622^ns^
No	87.30 (248,701)		86.30	1		
Yes	12.70 (36,120)		13.70	1.14 [0.67–1.93]	0.622^ns^	
Under 5 years old						0.016^*^
No	64.30 (183,075)		70.50	1		
Yes	35.70 (101,746)		29.50	0.67 [0.49–0.93]	0.016^*^	
50+ years old						0.058^ns^
No	84.70 (241,380)		81.50	1		
Yes	15.30 (43,441)		18.50	1.40 [0.99–1.99]	0.058^ns^	
Number of days lost to illness						0.023^*^
Zero	28.50 (19,973)		23.90	1		
Less than a week	27.10 (20,074)		24.00	1.08 [0.61–1.91]	0.785^ns^	
A week to less than a month	38.00 (34,494)		41.20	1.44 [0.95–2.16]	0.084^ns^	
One month or above	6.40 (9196)		11.00	3.14 [1.46–6.76]	0.004^**^	
Sector of health facility						0.004^**^
Public sector	61.90 (176,059)		71.60	1		
Private sector	26.60 (75,737)		23.80	0.69 [0.4–1.19]	0.182^ns^	
Informal sector	11.50 (32,843)		4.70	0.26 [0.15–0.46]	< 0.001^***^	
Total number of paid visits, mean (SE)	1.18 (0.02)		1.69	1.13 [0.96–1.33]	0.131^ns^	0.131^ns^
Share of OOP on drugs, mean (SE)	0.82 (0.02)		0.75	0.24 [0.08–0.67]	0.007^**^	0.002^**^
Health insurance status						0.577^ns^
Uncovered	98.97 (3251)		98.90	1		
Covered	1.03 (34)		1.10	0.80 [0.36–1.78]	0.577^ns^	
HF has functional X-rays						0.004^**^
No	99.90 (284,563)		99.90	1		
Yes	0.10 (257)		0.10	1.85 [1.21–2.83]	0.005^**^	
HF has functional ultrasound						0.036^*^
No	98.70 (281,249)		97.70	1		
Yes	1.30 (3571)		2.30	2.91 [1.02–8.29]	0.045*	
HF has functional microscopes						0.495^ns^
No	51.10 (145,409)		47.30	1		
Yes	48.90 (139,411)		52.70	1.24 [0.67–2.29]	0.495^ns^	
HF has functional sterilizers						0.306^ns^
No	52.50 (149,396)		46.90	1		
Yes	47.50 (135,425)		53.10	1.37 [0.75–2.52]	0.307^ns^	
HF has functional centrifuges						0.135^ns^
No	81.30 (231,472)		87.30	1		
Yes	18.70 (53,349)		12.70	0.54 [0.24–1.22]	0.139^ns^	
HF avg. price for care services, mean (SE)	5.00 (1.12)		7.72	1.01 [1–1.02]	0.188^ns^	0.188^ns^
Long travel time (30+ min)						0.006**
No	16.40 (46,632)		8.50	1		
Yes	83.60 (238,188)		91.50	2.64 [1.29–5.4]	0.008^**^	
HF level 1: Health center						0.357^ns^
No	41.80 (119,096)		38.30	1		
Yes	58.20 (165,543)		61.70	1.23 [0.79–1.93]	0.357^ns^	
HF level 2: General reference hospital					< 0.001^***^	
No	95.80 (272,754)		89.40	1		
Yes	4.20 (11,884)		10.60	7.73 [3.78–15.78]	< 0.001^***^	
Dissatisfaction index score						0.696^ns^
No dissatisfaction	17.90 (50,927)		15.00	0.81 [0.28–2.3]	0.686^ns^	
Score 1 Low dissatisfaction	23.10 (65,925)		22.60	0.99 [0.37–2.65]	0.979^ns^	
Score 2	23.20 (65,980)		27.40	1.31 [0.51–3.4]	0.573^ns^	
Score 3	16.30 (46,520)		16.70	1.05 [0.31–3.58]	0.934^ns^	
Score 4	12.10 (34,555)		11.10	0.91 [0.3–2.69]	0.858^ns^	
Score 5 High dissatisfaction	7.30 (20,914)		7.20	1		

Statistical significance of *: 0.01 ≤ p < 0.05; **: 0.001 ≤ *p* < 0.01; ***: p < 0.001; ns: p ≥ 0.05

All estimates are weighted

*HF* health facility

In the univariate logistic regression analysis (Table 4), demand-side factors such as the sector of preference for seeking care, severity of illness (estimated by the reported days lost), young and adult age (vs. under 5), geographic area, wealth and the share of OOPs on drugs were independently associated with higher OOPs (*p* < .05). For supply-side factors, having functional X-rays or ultrasound available in the health facility, long distance to care and the level of operation as a general reference hospital were significantly associated with higher OOPs (*p* < .05).

Further, from the sensitivity analysis with respect to the two alternative thresholds, we noted that the share of OOPs on drugs was significantly lower among those with extremely high expenditure on outpatient care compared to those who did not (*p* < .05). We also noted higher average prices for care among those with extremely high expenditure compared to those who do not (*p* < .05). Finally, those who travelled ≥30 min for care services were more likely to have higher expenditures than those who did not (*p* < .05).

### Multivariate logistic regression analysis

A multivariate analysis explored the predictors of excessive OOPs for outpatient care. We first present results from our main model using ≥2 times the median OOPs as a cut-off point for high expenditure. Then, we highlight key findings from the models using the two additional thresholds: above the median (“medium-high expenditure”) and ≥ 3 times the median OOPs (“extremely high expenditure”).

In the multivariate analyses (Table 5), we found that individuals in the lower wealth quintiles were significantly less likely than those in the wealthiest quintile to spend high OOPs on outpatient care (*p* < 0.05). The adjusted odds ratios (aORs) ranged from 0.37 in the poorest quintile to 0.49 in the wealthier quintile, compared to the wealthiest quintile. We also found that, ceteris paribus, having one’s own means of transportation had a protective effect against excessive expenditure in this population (aOR 0.43, *p* < 0.05). By contrast, we found that individuals in households with six or more members were 1.8 times more likely to spend at least twice the median OOPs (*p* < 0.05). We also found that, the likelihood of excessive expenditure was 3.97 times higher among those who stopped their usual activity for one month or longer, compared to individuals who did not lose any days of activity due to the illness or injury (*p* < 0.05). Interestingly, we found that increases in the share of OOPs on drugs and medications was associated with a lower likelihood of incurring excessive costs (aOR 0.21, *p* < 0.05). We also found that treatment costs for children under age five were less likely to be excessive (aOR 0.58, *p* < 0.05) after controlling for other factors, compared to treatment costs for older individuals. Moreover, smaller households, living in the Maniema or Orientale provinces, and reporting an elevated dissatisfaction with services received (≥4 user complaints) were associated with higher likelihood of incurring excessive expenditure. Among the variables entered in the model to capture the technological capacity of the health facility for disease diagnosis, going to a facility with at least one functional ultrasound machine increased the likelihood of excessive OOPs (aOR 3.84, *p* < .10). Finally, we found that individuals who went to a general reference hospital were 50.6 times more likely than those who did not go to a general reference hospital to incur excessive costs (*p* < .05). Other factors were insignificant. With respect to prices charged in facilities, we found an interesting finding showing that the association was insignificant in the multivariate model.

**Table 5:** Multiple logistic regression analysis: relative influence of demand- and supply-side factors on the likelihood of incurring excessive out-of-pocket expenditure (OOPs) for outpatient care

Determinant	Adjusted OR	SE	*p*-value
Poorest (vs. Wealthiest)	0.37	0.16	0.023^*^
Poorer	0.44	0.16	0.022^*^
Middle	0.46	0.16	0.031^*^
Wealthier	0.49	0.14	0.012^*^
Highest education of hh head: Primary (vs. None)	1.45	0.80	0.500^ns^
Secondary	1.63	0.75	0.291^ns^
Tertiary	1.03	0.68	0.963^ns^
Owns a transportation mean	0.43	0.12	0.002^*^
Large household size (≥6)	1.77	0.50	0.042^*^
Days lost: Less than a week (vs. Zero)	1.27	0.40	0.441^ns^
Days lost: A week to less than a month	1.56	0.48	0.152^ns^
Days lost: One month or above	3.97	0.19	0.005^*^
Private sector (vs. Public sector)	4.93	8.36	0.348^ns^
Informal sector	2.50	4.18	0.584^ns^
Total number of paid visits	0.91	0.14	0.570^ns^
Share of OOPs on drugs	0.21	0.12	0.007^*^
Equateur (vs. Maniema/Orientale)	0.23	0.09	0.000^***^
Kasai Occidental/Oriental	0.37	0.14	0.011^*^
Male (vs. Female)	1.28	0.24	0.177^ns^
Head of household	0.85	0.38	0.722^ns^
Under 5 years old	0.58	0.11	0.003^*^
50+ years old	1.16	0.38	0.658^ns^
HF has functional X-ray	3.03	1.92	0.081^ns^
HF has functional ultrasound	3.84	1.98	0.010^ns^
HF has functional microscopes	1.13	0.43	0.747^ns^
HF has functional sterilisers	1.12	0.42	0.770^ns^
HF has functional centrifuges	0.35	0.16	0.021^*^
HF avg. price for care services	1.01	0.00	0.085^ns^
Long travel time (30+ min)	1.47	0.55	0.304^ns^
HF level 1: Health center	8.82	14.29	0.181^ns^
HF at Operational level 2 (Hôpital Général de Référence)	50.59	77.19	0.011^*^
Lowest dissatifaction (index score 1) (vs. No dissatisfaction)	1.98	0.73	0.063^ns^
Dissatisfaction index score 2	1.90	0.66	0.063^ns^
Dissatisfaction index score 3	1.98	0.78	0.082^ns^
Dissatisfaction index score 4	2.90	1.22	0.013^*^
Highest dissatifaction (index score 5)	2.52	1.37	0.090^ns^
Constant	0.24	0.49	0.485^ns^

Statistical significance of *: 0.01 ≤ p < 0.05; **: 0.001 ≤ p < 0.01; ***: p < 0.001; ^ns^: p ≥ 0.05;

*HF* health facility

With the sensitivity analysis, most of these results were fully consistent across all regression models that were estimated. This suggests that individuals belonging to lower wealth quintiles, owning a means of transportation, with a lower severity of illness (< 1 Month of days lost due to illness), living in the western provinces (Equateur, Kasai Occidental or Kasai Oriental) rather than in the northeastern ones (Maniema or Orientale), being a child under 5, and seeking care in health facilities not equipped with centrifuges seemed to be better protected against excessive OOPs. By contrast, education played a role only for the lowest threshold set for “medium-high” (greater than or equal to the median) expenditure, in which it showed a protective effect. In other words, when the household head completed at least primary education, individuals were less likely to incur higher OOPs (aOR were ranged from 0.30 to 0.40), compared to those in households where the household head did not complete primary education. The effect sizes of the operational level of care provision revealed some interesting patterns. While using health facilities delivering the complementary benefit package was significantly and positively associated with “high” (aOR 50.60) or “medium-high” OOPs (aOR 23.70), this association did not remain significant with “extremely high” (≥3 times the median) OOPs. On the other hand, going to health facilities delivering the minimal benefit package was significantly and negatively associated with “extremely high” OOPs (aOR = 0.50), but was not significant for the other models, suggesting that there could be a ceiling effect protecting individuals from extremely high OOPs.

## Discussion

Primary health care has been recognized as contributing to the effectiveness, efficiency, and equity of health services [24]. Although an orientation towards primary care would likely significantly reduce burden of disease, and as a result, the financial burden on households, most studies on out-of-pocket health care expenditure do not emphasize the role of primary care costs in contributing to catastrophic health expenditure. Yet, the implementation of quality modalities inherent to the three inter-related functions of health financing –revenue collection, fund pooling and purchasing/provision of services– will make the success of the UHC strategy. Among the many challenges is the mobilization and pooling of sufficient resources from the informal sector (> 70% of the population). One major challenge lie in bridging the gap between policy aspiration and practice, another in integrating the complex reality of the final beneficiaries. From a users’ perspective, we explored the extent to which Congolese households experience financial risk for outpatient services and the drivers of the direct burden.

Our study finds that one-third of individuals who were reported to have an illness or an injury in the four-week period prior to the survey did not seek out health care services. For those who needed primary care and did not use it, money was reported to be the primary reason for not seeking care. Although primary care is presumed to be used more by the poor, empirical evidence from sub-Saharan Africa suggests that the benefits to the poor are only marginally higher [25]. Our findings tend to corroborate the inverse care law phenomenon [26], as individuals in the poorest quintile were three times more likely to forego care than individuals in the wealthiest quintile. With respect to physical accessibility, important travel time to care provider was shown in our study for most patients. A study in South Africa used cost analysis combined with geographical information to explore primary care accessibility and utilization by rural population [27]. Their model allowed predicting more than 90% of utilization patterns, which may support its role in identifying deficiencies in coverage or rationalizing care supply. The very first contact with a care provider is therefore crucial (with respect to quality care, provider’s communication, availability and affordability of care) to facilitate effective coverage, continuity of care and treatment success. Following this idea, being at risk for catastrophic expenditure may jeopardize the likelihood of receiving timely appropriate care. Yet, our study results suggest that the poorest made more use of user fee exemptions and in that sense benefit from the pro-poor delivery of care strategy. Nevertheless, the financial constraint associated with primary care utilization was reported as the most common reason for not seeking or delaying care in all five wealth quintiles.

For individuals who used front-line services, nearly a third were at tangible risk of incurring catastrophic expenditure, as measured by having OOPs above a designated threshold. In particular, the cost burden of incurring high OOPs was concentrated among households in the top wealth quintile of the sample. Individuals belonging to poorer wealth quintiles tend to be better protected against high or extreme OOPs in absolute terms, although we were unable to quantify these expenditures as a proportion of total household expenditure (i.e., including non-health expenditure) due to the lack of household consumption expenditure data. However, it should be recognized that poorer households may have been priced out of the health care market, and individuals in these households may have chosen to forgo care altogether as a result.

Known for being one of the first sub-Saharan countries that established a primary health care and referral system, the DRC has a vast network of first-level health facilities distributed in health zones. Primary care is delivered through government facilities and private not-for-profit facilities under public contract which rely heavily on out-of-pocket contributions. The health economic literature provides ample evidence that user fees can deter service utilization, but drew attention to the need to remain cautious about the extent to which care usage can be affected by out-of-pocket contributions, or about the type of care concerned by reduced access induced by the presence of user fees [28].

In spite of the above, providing effective primary health care is challenging due to inequities in access, utilization and payments for services and idled decentralization [6, 29]. We found that inequities in financial protection were highly pronounced when measured by the location of care services. In addition to patient characteristics, our results attributed a substantial share of the financial burden associated with the use of primary health care to various factors related to territorial access to and provision of health care services. Discrepancies across geographic areas suggest that individuals residing in the northwest provinces and, with limited evidence, urban areas tend to be at higher risk of high and extreme spending. Discrepancies between tertiary and lower level health facilities had a significant impact on whether consumers effectively incurred high or extreme levels of OOPs. Moreover, in some regions, public service provision failed for the benefit of the informal sector [12], which was not shown as critical in our study.

Regarding medical pluralism, despite that Congolese users tend to primarily rely on state-owned health facilities, potential overuse of private care consultations combined with traditional medicines can still be raised from our findings and was shown in Goma, DRC, among the elderly [30]. In the latter study, predictors of seeking care outside of the public sector related to quality of public care and household wealth among other access barriers to public care services including the lack of financial protection for health [30]. With respect to informal care, evidence suggests that it is likely used to complement rather than substitute modern care [30], which is in line with our results. We also found that the most important component of OOPs for outpatient care were fees for drugs and medication. This observation highpoints the necessity of improving access to medicines from a health system perspective. More generally, few studies have looked at patients’ cost burden of outpatient care. According to recent evidence from National Health Accounts analysis, sub-Saharan African households spent on average 11 dollars per capita on primary health care (PHC), which is a higher number but rather consistent number with our findings, especially in urban areas [31]. Addressing catastrophe requires the availability of household expenditure data to inform the health financing policy process, which remains scanty in fragile states such as the DRC [32].

A key rationale to introduce or extend health insurance programs is to broaden risk pooling. We found that voluntary enrolment to community-based insurance schemes was highly regressive, which looks consistent with the literature in the field [25]. Besides, among the countries that implemented health insurance reforms, some were effective in reducing out-of-pocket expenditure while other had smaller impact or even increased out-of-pocket expenditure [33]. In average, our findings suggest increased out-of-pocket expenditure under an employer-offered plan, compared to expenditure reported by individuals without insurance coverage. At the opposite, these tend to be reduced when the care user is covered by a mutual health organization. Yet, risk-sharing and its impact on improved equitable access to the needed services or on reduced catastrophic health expenditure are very limited [28] – even more striking if one seeks to show the impact for outpatient (excluding inpatient). When available, evidence often focus on enrolment issues rather than effective benefits from pooled and prepayment mechanisms. Actually, our multivariate regression outputs did not reveal a statistically significant impact of being covered by an insurance plan with respect to financial health risk.

Furthermore, the context in which risk-sharing is implemented matter greatly [28]. For the purpose of scaling up health care coverage, Mexico established a vast program of public insurance. The context is different but this study has the merit of having documented that the risk-sharing mechanism can be effective to lower financial risk for beneficiaries [34]. However, echoing our finding, the reduction in catastrophic expenditure varied greatly according to supply-side parameters such as the type of health facilities. Although the context is not replicable, these results would support to test a strategy that place a greater supply of well-trained/paid care providers and services in the critical zones [35].

Likewise, an interesting finding from a study in rural China showed that implementing a new scheme of benefit package for outpatient care may contribute to changes in billing practices of insured patients. In our settings, less than 4% were covered by a health insurance plan. However, regardless health insurance type of affiliation, reported expenditure tended to be greater among insured patients. Nevertheless, our findings suggested that it is not evident that excessive OOPs can be attributable to posted prices at facilities. Although price responsiveness may remain contentious [36–39], a recent study, which was conducted in the same Congolese provinces, indicated that health care consumers tend to be insensitive to prevailing prices of curative care in health facilities [40]. Marginal changes in prices had relatively minimal effects on the use of outpatient services, which suggests that potential change in charging practices would exacerbate the direct financial burden of primary care. However, that study ran analysis on rural areas only and excluded urban beneficiaries. Future studies should investigate whether price elasticity of demand may take a different form in urban areas. Even if demand for primary care tends to be inelastic to prices, it is of high importance to further look at fees health facilities run. For example, data from a related health worker ASSP study revealed that health staff may not be paid on time (i.e., they have not been paid for months), so one could expect a change in how providers cope with low resources and possibly an adverse effect on the quality of care. Besides, the literature in the field suggests that an unregulated fee-for-service payment system, as it prevails for outpatient care in the DRC, may induce higher patients’ costs at district hospitals than at health centers [12]. Also, greater financial barriers to care were reported at general hospitals compared to first-level facilities, which aligns with our findings.

Furthermore, our findings present a conservative estimate of OOPs, as under-the-table or extra payments in addition to official listed prices may have been made, but not have been explicitly reported. Nonetheless, fraud and malpractices inherent to a lack of knowledge of the current financing system have been observed in some health centers in Kisantu District, DRC, particularly through staff selling referral bills to patients or charging patients for items already covered by a flat fee [12]. In Africa, evidence suggests a robust correlation between the level of (in)formal payments and health system failures–even if causality remains to be assessed [2, 41]. Price discrimination practices may occur to overcome the absence of nation-wide cross-subsidization from wealth to poor (income) [41]. On the other hand, coping with relatively ineffective ‘EXIT and VOICE strategies [42]’ as ultimate mechanisms to enhance providers’ responsiveness may also exacerbate the burden [43]. In several respects, the situation is distressing and would require a superior focus on tackling health system failures that create the economic burden of illness. Among other difficulties in DRC, a recent study looking at human resources for health financial remuneration [1] found that informal sources of income accounted for a significant proportion of health workers income, in addition to stipulated user fees. More specifically in the studied provinces, the related health worker survey showed that 73% of health staff reported having received income directly from user fees; and when they were asked if their remuneration from the salary they were supposed to be receiving adequately covered their basic needs, about 80% of health staff reported not being able to cover their basic needs. In line with, another study in the DRC estimated monthly wages of civil servants ranges from $15 to $30, where family consumption expenditure for basic needs such as food, housing, transportation and education may go up to ten times this estimation [29].

Finally, a recent study in Vietnam showed that maternal care seekers who achieved the most appropriate level of care were at the same time not the most efficient at using these services [44]. These authors introduced the concept of demand-side efficiency i.e., “the efficiency with which health system users convert public health resources into health outcomes” and claim for technical efficiency estimation from the users’ perspective to inform policy-makers. To relate to inequities shown in the DRC, such evidence may be useful to further examine how availability of care at iso-resources for all can lead to different outcomes and secure appropriate use of outpatient care services. Also, patients’ satisfaction with the health care delivery system (“perceived quality of care”) as a driver of erratic care pathways and generator of extra-costs remains of interest for implementers searching for effective policies. Our findings highlighted that elevated dissatisfaction lead to higher risk of incurring excessive expenditure. From a system perspective, reallocating government subsidies in primary care has been identified as a lever to improve health system efficiency and equity [25].

### Study strengths and limitations

A strength of the study was the use of a wide range of demand- and supply-oriented variables and the use of a population-based household survey of reported patients’ costs for primary health care in the DRC. Such population-based studies can have important implications for the design and implementation of ongoing health financing reforms, and more specifically, the development of a sustainable public financial protection strategy over the years to come.

The findings of this study should be interpreted within the context of several limitations. First, due to the complexity of collecting sensitive data such as direct costs in low-income settings, misclassification, under-reporting of small or nonmedical costs categories, or misreporting of lump sum items may have occurred. These are unavoidable issues in patients’ cost studies and have been recognized as producing minor effects on cost [45]. On the other hand, capitalized knowledge as part of the research project has facilitated quality data standards. Second, data on household consumption/expenditures was not available, which prevents the calculation of catastrophic health expenditures and the financial burden of the specified levels of OOPs. In addition, our study only provides an overview of the determinants of OOPs for outpatient care as a whole, and more studies may be needed, disaggregating OOPs by type of care or disease. Third, the independent variables investigated included the type of health facility first visited when they sought care, but this should not be an issue, as the mean number of visits averaged one visit per individual. This suggests further studies to look at the different care options in order to determine the extent to which costs might be prohibitive and extend the data collection period beyond the four-week period used in this study. In addition, another measure of price than averages the prices charged by health facility could be used in future research. Fourth, we used patients’ satisfaction with the care they received as a proxy for the facility’s quality of health care based on the available data, but future studies should consider refining the measure to better capture the full complexity of the quality care-delivery construct. Finally, evidence reported here purposely focused on outpatient care to help the UHC development process. These findings therefore do not present the full picture of risk of impoverishment from seeking medical care, as inpatient expenditures, which are often much higher than outpatient expenditures, were not included from the OOPs calculation. However, individuals are also more likely to seek outpatient care instead of inpatient care, so the findings of this study remain timely and relevant for the Congolese UHC conversation.

## Conclusion

Outpatient expenditure, as a component of the overall direct expenditure for health, exacerbates the financial risk incurred by the households in the DRC. Using population-based estimates, our paper contributes to the empirical literature in three main areas.

First, assessing population-based patterns and determinants of the likelihood and financial burden of incurring direct expenditure for outpatient care yielded evidence on households’ willingness to pay for primary care and basic unmet needs, which are essential pillars for developing prepayment models for care based on national insurance funds that are currently under discussion. With less than 10% of the urban population covered by formal health insurance programs – and even less for the rural population – the fact that the national health system heavily relies on households’ direct contributions and primary care constitutes the entry point into the system.

Second, disaggregated data on the medical and nonmedical types of expenditure may also improve understanding of the nature of the economic and social hardships experienced by households. For instance, even though drugs and medication accounted for a large proportion of primary health care spending, it was not a main predictor of excessive outpatient bills in the Congolese context, whereas other costs, such as user fees charged by health providers at the point of care, may be more important. Evidence on how outpatient expenditure is distributed in a population-based setting, its magnitude and the main predictors of catastrophic expenditure should engage health stakeholders to better plan the distribution of available resources. New evidence should help to design preferred care delivery strategies and population targets (general population and vulnerable patient subgroups), as well as to plan care delivery options at the most appropriate level of the health care pyramid (operational and more central level of care delivery).

Finally, ranked as the seventh most fragile state in 2016 [46], provision of public services in the DRC requires effective policing towards improved health equity and service coverage. Multiple wealth-related disparities were shown in our study. Strong primary care services, as part of the universal health coverage strategy, can cover many of the population needs, and measuring it matters. Our study of the patients’ costs for outpatient care attempted to inform technical and political decisions related to the current financing reform. Realizing equitable access to primary care through financial protection mechanisms must be a vital building block for the universal health coverage reform. In particular, sustainable financing to promote efficient access to care is a challenge in all countries and should be a vital matter in resource-poor countries.

## Abbreviations

AfHEA: African Health Economics and Policy Association
ASSP: Accès aux Soins de Santé Primaires [Access to primary health care]
CNP-SS: Comité National de Pilotage du Secteur de la Santé [General Assembly of the National Steering Committee of the Health Sector]
DHS: Demographic and Health Survey
DPS: Divisions Provinciales de la Santé [Provincial Health Divisions]
DRC: Democratic Republic of Congo
EU: European Union
I^3^h: Institute for Interdisciplinary Innovation in Healthcare
IRB: Institutional Review Board
OOPs: Out-of-pocket expenditures
OR: Odd Ratio / aOR adjusted Odd Ratio
p: probability
PCA: Principal Component Analysis
PPS: Probability Proportional to Size
SD: Standard Deviation
SE: Standard Error
UHC: Universal Health Coverage
USA: United States of America
WHO: World Health Organization
